# The Smad3-dependent microRNA let-7i-5p promoted renal fibrosis in mice with unilateral ureteral obstruction

**DOI:** 10.3389/fphys.2022.937878

**Published:** 2022-08-25

**Authors:** Ze Peng, Huai-Ying Guo, Yu-Qing Li, Jian-Chun Li, Xiao-Hong Yang, Jian Liu, Qiong-Dan Hu, Hong-Lian Wang, Li Wang

**Affiliations:** ^1^ College of Integrated Chinese and Western Medicine and the Affiliated Traditional Chinese Medicine Hospital, Southwest Medical University, Luzhou, China; ^2^ The Clinical Laboratory of the Affiliated Traditional Chinese Medicine Hospital, Southwest Medical University, Luzhou, China; ^3^ Research Center for Integrative Medicine, The Affiliated Traditional Chinese Medicine Hospital of Southwest Medical University, Luzhou, China; ^4^ The Department of Nephrology of the Affiliated Traditional Chinese Medicine Hospital, Southwest Medical University, Luzhou, China; ^5^ School of Clinical Medicine, Chengdu University of Traditional Chinese Medicine, Chengdu, China

**Keywords:** let-7i-5p, UUO, renal fibrosis, Smad3, CRISPR/Cas13d

## Abstract

Renal fibrosis is a common feature of all types of chronic kidney disease (CKD) and is tightly regulated by the TGF-β/Smad3 pathway. Let-7i-5p belongs to the let-7 microRNA family with diverse biological functions. It has been reported that let-7i-5p suppresses fibrotic disease in the heart, lungs, and blood vessels, while the role of let-7i-5p in renal fibrosis remains limited. In this study, we aimed to investigate the role of let-7i-5p in renal fibrosis in a mouse model of unilateral ureteral obstruction (UUO) and TGF-β1–stimulated renal tubular cell line TCMK1. The RNA-targeting CRISPR/Cas13d system was used to knock down let-7i-5p. Renal injury and fibrosis were determined by histological analysis, RT-PCR, Western blot, and immunostaining. Our results have shown that in the kidneys after UUO, the expression of let-7i-5p was significantly increased along with notable tubular injury and interstitial fibrosis. Electroporation of let-7i–targeting Cas13d plasmid efficiently knocked down let-7i-5p in kidneys after UUO with reduced tubular injury, fibrotic area, and expression of fibrotic marker genes α-SMA, fibronectin, and Col1a1. In TGF-β1–stimulated TCMK1 cells, knockdown of let-7i-5p by Cas13d plasmid transfection also blunted the expression of fibrotic marker genes. Most importantly, the genomic locus of let-7i showed enriched binding of Smad3 as revealed by chromatin immunoprecipitation. In TCMK1 cells, the overexpression of Smad3 can directly induce the expression of let-7i-5p. However, the deletion of Smad3 abolished TGF-β1–stimulated let-7i-5p expression. Collectively, these findings suggest that let-7i-5p is a Smad3-dependent microRNA that plays a pathogenic role in renal fibrosis. Let-7i-5p could be a promising target for the treatment of CKD-associated renal fibrosis.

## Introduction

The global incidence of chronic kidney disease (CKD) is about 13% which casts a non-negligible burden on the healthcare system (Evans et al., 2022). Renal fibrosis is the result of over-healing of wounds after injury and is characterized by unwanted deposition of extracellular matrix (Gu et al., 2020). Renal fibrosis is a common feature of chronic kidney disease (CKD) and also positively correlates with the deterioration of renal function in CKD (Gu et al., 2020). Therefore, targeting fibrosis is a promising option to ameliorate CKD, which, however, largely relies on the resolution of the mechanism underlying renal fibrosis.

TGF-β signaling is the master regulator of renal fibrosis (Wang et al., 2021). The canonical TGF-β signaling is initiated by the binding of TGF-β ligand to membrane receptors TGFBR1/2 followed by the activation of cytosolic Smad2 and Smad3 *via* phosphorylation. Phosphorylated Smad2/3 translocate to the nucleus to modulate target gene transcription (Wang et al., 2021). It is now recognized that Smad3 but not Smad2 is the primary transcriptional factor mediating renal fibrosis in various types of CKD (Wang et al., 2021).

MicroRNAs are a group of noncoding RNAs of about 22 nucleotides in length and execute versatile biological roles in physiopathology. By binding to the 3′ untranslated region (UTR), microRNAs function to degrade or inhibit the translation of the target mRNAs (Bartel, 2004). Let-7 is the first microRNA identified in *Caenorhabditis elegans*. Later on, a group of let-7 family members have been found in vertebrates with evolutionary conservation (Roush and Slack, 2008). For example, there exist 11 member genes in the let-7 family of mice and humans (Roush and Slack, 2008). The miR-let-7 family plays important roles in development, proliferation, tumorigenesis, and differentiation (Roush and Slack, 2008). Among them, let-7i is involved in organ fibrosis. In the cardiovascular system, it has been reported that let-7i-5p attenuates angiotensin II–induced cardiac fibrosis by targeting several collagen-coding genes such as *Col1a2*, *Col3a1*, *Col4a1*, and *Col5a2* (Wang et al., 2015). Let-7i also suppresses endothelial–mesenchymal transition and cerebral vascular fibrosis after ischemic stroke (Chen et al., 2020). In the lung fibroblasts, mesenchymal stem cell–derived let-7i-5p inhibits fibroblast activation and ameliorates pulmonary fibrosis in silicosis disease (Xu et al., 2022). In contrast to the findings in the cardiovascular system and lungs, emerging evidence shows that let-7i-5p is upregulated in CKD and may aggravate renal fibrosis (Jin et al., 2021; Sun et al., 2021). Despite these signs of progress, more evidence is warranted for a convincing role of let-7i-5p in renal fibrosis. Furthermore, it is still not clear how let-7i-5p is regulated in renal fibrosis.

Since its first emergence, the CRISPR/Cas technology has rapidly developed to realize the edition of DNA and RNA both *in vitro* and *in vivo* (Pickar-Oliver and Gersbach, 2019). Cas13d belongs to the class 2 type VI CRISPR/Cas effector which can be used for RNA-targeting degradation or knockdown. Among them, Cas13d from the *Ruminococcus flavefaciens* XPD3002 (designated as RfxCas13d) can knock down target RNA with superior efficiency and specificity when compared with the conventional RNA interference–based technologies (Konermann et al., 2018).

In this study, we applied Cas13d-mediated gene knockdown to study the role of microRNA let-7i-5p in renal fibrosis. We found that let-7i-5p knockdown attenuated fibrosis in UUO mice and TGF-β1–treated renal tubular cells (TCMK1). Furthermore, we also discovered that let-7i-5p is a target gene of the pro-fibrogenic TGF-β/Smad3 pathway.

## Materials and methods

### Plasmids

The CRISPR-Cas13d vector targeting pri-let-7i was constructed by cloning the guide RNA sequence (5′-ACT​CCA​TCA​TCA​AAC​ACG​ACA​A-3′, which is complementary to the sequence of mature let-7i-5p) under U6 promoter of the backbone vector pLVX-U6-RfxCas13d (purchased from Haijihaoge Biotech., China). The recombined vector was designated as pCas13d-let-7i for short. The Smad3 overexpression plasmid pcDNA3.1-Smad3 was previously described in Wang et al. (2022).

### Animal experiment

Male C57BL/6J mice, 8–10 weeks old, were purchased from GemPharmatech Co., Ltd. (Chengdu, China). The mice were adaptively fed for 1 week. Then, unilateral ureteral obstruction (UUO) or sham operation was performed on the right kidney as previously described (Ji et al., 2018). Seven days after the operation, the mice were sacrificed by overdose of pentobarbital sodium and the kidneys after UUO or sham operation were harvested. For histological analysis, a part of the kidneys was fixed in 4% paraformaldehyde followed by standard paraffin embedding. For the Western blot and RT-PCR, the medullary part of the kidneys was removed and the remaining cortex was used for isolation of protein and RNA. All animals were kept in a specific pathogen-free (SPF) facility with a 12-h light/dark cycle, with free access to food and water. All animal experimental manipulations were approved and in compliance with corresponding regulations issued by the Animal Ethics Committee of Southwest Medical University (Ref. No. 20211119-019).

### Gene delivery in kidney by *in situ* electroporation

The electroporation-mediated gene delivery was performed 1 day before the UUO operation. The animal was anesthetized with pentobarbital sodium. After shaving the dorsal hair, an incision (of about 0.5 cm) was made to expose the right kidney. Then, the renal artery and vein were clamped with an arterial clamp. Then, 100 μg of let-7i-5p knockdown plasmid (pCas13d-let-7i in 100 μl saline) was slowly delivered into the kidney by retrograde renal vein injection through a 31-G needle. Electroporation was performed with the CUY21EDIT II Electroporator (BEX Co., Japan). Briefly, the kidney was clamped with a pair of tweezer-like electrodes. An electronic pulse of 50 V was applied four times. After electroporation, the artery clamp was removed and the kidney was replaced into the abdominal cavity. The incision was sealed, and the animal was placed in a warm place, waiting to wake up. For the sham operation, only 100 μl of saline was injected, followed by electroporation. The electroporation efficiency in the kidney was monitored by electroporating a plasmid coding the enhanced green fluorescent protein (EGFP). Electroporation following the aforementioned parameters can realize ubiquitous EGFP expression in the cortex tubules (Supplementary Figure S1).

### Histological analysis

The paraffin-embedded kidney tissue was cut into 4-μm-thick sections. Hematoxylin and eosin (HE) staining and Masson staining were performed as previously described (Ji et al., 2018). The tubular injury score was calculated based on the percentage of injured tubules as previously described (Yang et al., 2021). The fibrotic area was defined as the area of blue collagen fiber revealed by Masson staining.

### Cell culture

The mouse renal tubular cell line TCMK1 was purchased from ATCC (cat# CCL-139, United States) and cultured in DMEM (BasalMedia, cat# L110KJ, China) + 10% fetal bovine serum (FBS, Gibco, cat# 16000-044, United States) + 1% penicillin–streptomycin (Beyotime, cat# C0222, China) at 37°C with 5% CO_2_ and 100% humidity. All experimental treatments were performed in the 6-well plate. For the treatment with TGF-β1, TCMK1 cells of 80%–90% confluence were starved in the medium containing 0.5% FBS for 12 h. Then, 5 ng/ml of TGF-β1 was added to stimulate for the indicated time. For plasmid transfection, TCMK1 cells of about 50% confluence were transfected with the indicated plasmid (4 μg in each well) by polyethylenimine reagent. The medium was replaced 24 h after transfection.

### Reverse-transcription polymerase chain reaction

Total RNA of the kidney tissue or TCMK1 cells was isolated with the TRIzol reagent (Tiangen, cat# DP424, China). For the detection of protein-coding gene and U6, 1 μg of RNA was used for cDNA synthesis with random hexamers by the HiScript III 1st Strand cDNA Synthesis Kit (Vazyme, cat# R312, China) followed by PCR amplification with Taq Pro Universal SYBR qPCR Master Mix (Vazyme, cat# Q712, China). For the detection of let-7i-5p, cDNA was synthesized from 1 μg of RNA with the let-7i-5p–specific stem-loop primer (5′-GTC​GTA​TCC​AGT​GCA​GGG​TCC​GAG​GTA​TTC​GCA​CTG​GAT​ACG​ACA​ACA​GC-3′) by the miRNA 1st Strand cDNA Synthesis Kit (Vazyme, cat# MR101, China) followed by PCR amplification with miRNA Universal SYBR qPCR Master Mix (Vazyme, cat# MQ101, China). The relative expression of an indicated gene is calculated by the 2^−∆∆Ct^ method. The expression levels of mRNA-coding genes were normalized with GAPDH while the expression level of let-7i-5p was normalized with U6. Primers used in PCR amplification are listed in Supplementary Table S1.

### Western blot

Protein was isolated with the RIPA lysis buffer and quantitated by the BCA method. Then, 20 μg of protein was separated in 10% SDS-PAGE gel and transferred to the PVDF membrane. After blocking with 5% fat-free milk powder, the indicated primary antibody was applied to incubate at 4°C overnight. For the detection of phosphorylated protein, 2.5% bovine serum albumin (BSA) was used for blocking and primary antibody incubation. The next day, the membrane was washed with TBST and incubated with HRP-conjugated secondary antibody at room temperature (RT) for 1 h. After washing with TBST, signals were developed with ECL Western Blotting Substrate (Affinity, cat# KF8005, United States) and visualized by ChemiScope 600 EXp system (ClinX, China). ImageJ 1.47v software (NIH, United States) was used to quantitate the relative expression of the indicated protein by normalizing it to GAPDH. Antibodies used in Western blot are listed in Supplementary Table S2.

### Immunohistochemistry

The paraffin tissue section was dewaxed in xylene and rehydrated in gradient ethanol solution. Antigen retrieval was performed in 10 mM citrate solution (pH 6.0) by boiling in a microwave oven for 10 min. Endogenous peroxidase was blocked by treatment with 3% H_2_O_2_ for 15 min at RT. After further blocking with 2.5% BSA, the primary antibody was applied to incubate at 4°C overnight. The next day, after washing with PBS, the downstream manipulations were performed with the Universal Mouse/Rabbit HRP-conjugated Polymer Detection Kit (ZSGB-Bio, cat# PV-6000, China) following the manufacturer’s instruction. DAB was used for chromogenesis. Images were taken with a light microscope (Leica, ICC50W, Germany).

For immunofluorescence, the OCT-embedded fresh kidney tissue was cut into 4-μm-thick sections and fixed with 4% paraformaldehyde for 10 min. After permeabilization with 2.5% Triton X-100 for 15 min, the section was blocked with 2.5% BSA for 1 h. The primary antibody was incubated at 4°C overnight. The next day, the section was washed with PBS and incubated with fluorescent secondary antibody for 1 h. The nuclei were counterstained with DAPI. Images were captured with a fluorescent microscope (EVOS FL Auto Cell Imaging System, Invitrogen, United States). Antibodies used in immunohistochemistry are listed in Supplementary Table S2.

### Chromatin immunoprecipitation

TCMK1 cells were seeded into the 10-cm culture dish. When the confluence reached 90%, the cells were starved in a medium with 0.5% FBS for 6 h followed by stimulation with 5 ng/ml TGF-β1 for 30 min. The cells were then harvested for chromatin immunoprecipitation (ChIP) assay with a rabbit anti-Smad3 antibody (CST, cat# 9523, United States) with the Magnetic Bead ChIP Kit (Pierce, cat# 26157, United States). The chromatin precipitated by the Smad3 antibody was subjected to DNA purification which was used for PCR analysis (ChIP-PCR) or sequencing (ChIP-seq). ChIP-seq and downstream bioinformatic analysis was performed as previously described (Sheng et al., 2021). The distribution of Smad3 binding peaks in the genomic locus of let-7i was visualized by UCSC Genome Browser. The raw data for ChIP-seq is accessible in the Gene Expression Omnibus database (GSE203063).

### Smad3 knockout TCMK1 cells

The Smad3 knockout (Smad3KO) TCMK1 cells were generated with CRISPR/Cas9-mediated gene knockout. Briefly, TCMK1 cells were transfected with the vector pX330-Smad3 which co-expressed Cas9 protein and mouse Smad3-targeting gRNA (with gRNA target sequence of 5′-GTC​CCC​AGC​ACA​CAA​TAA​CT-3′). Besides, this vector also co-expressed EGFP. After transfection, the EGFP-tagged cells were sorted with flow cytometry (BD Bioscience, FACSAria III, United States) and subjected to single cell–derived clonal expansion. DNA fragment flanking the edition site of the Smad3 locus was amplified by PCR in those successfully expanded cell clones and analyzed by Sanger sequencing (Sango, China). Cell clones with presumable deletion of Smad3 were further validated by Western blot analysis.

### Statistics

All quantitative data were presented as mean ± standard derivation (SD). A student’s *t*-test was used for comparison between two groups while one-way ANOVA with LSD test was applied for multigroup comparison. *p* < 0.05 was regarded as statistically significant. GraphPad Prism 5 software was used for statistical analysis and graph drawing.

## Results

### Unilateral ureteral obstruction–induced renal fibrosis was accompanied by increased let-7i-5p expression

**FIGURE 1:**
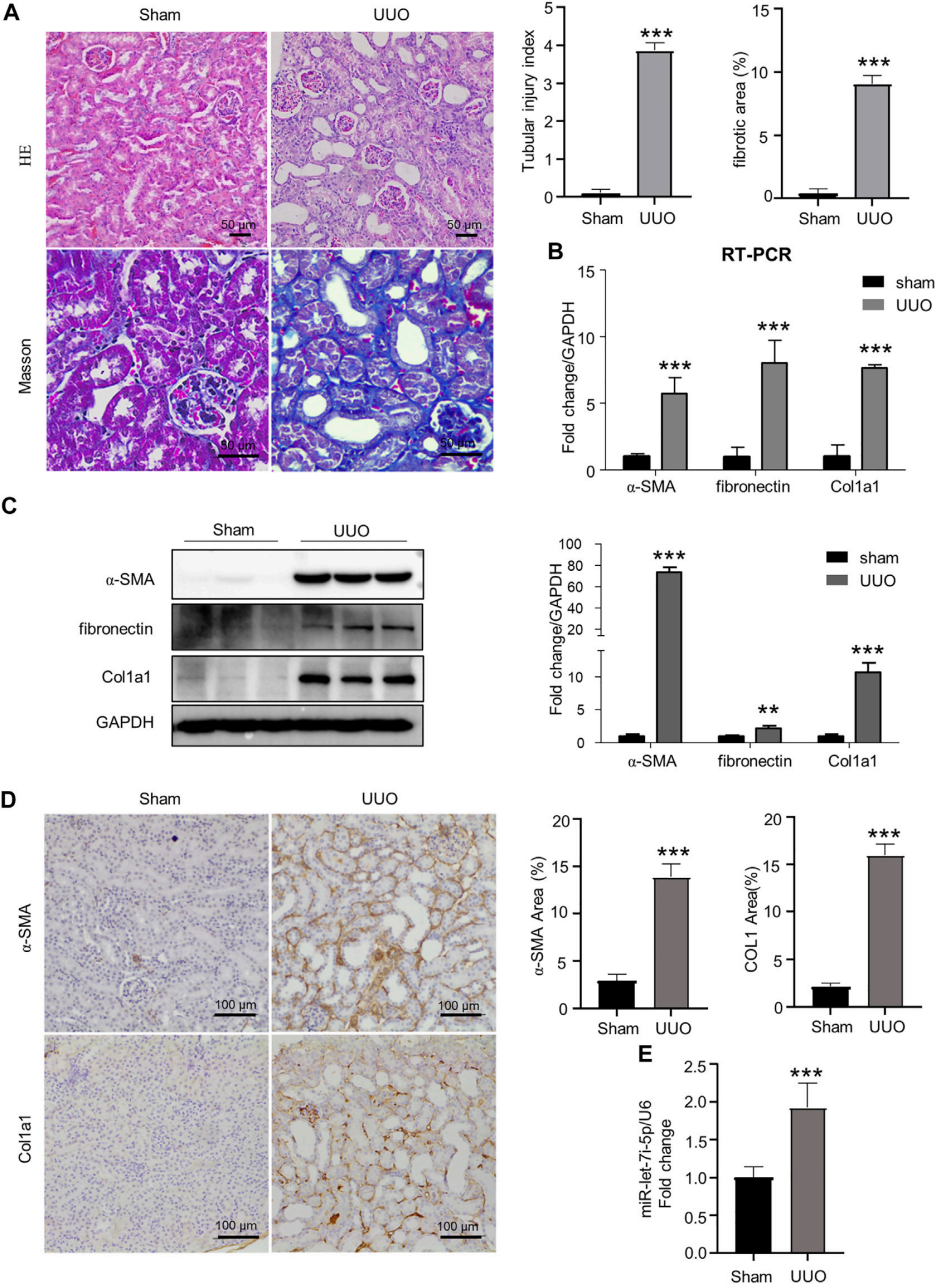
UUO causes renal fibrosis with increased let-7i-5p expression. **(A)** HE and Masson staining of the kidney tissue. The graphs on the upper right are quantitation of tubular injury index and fibrotic area based on HE and Masson staining, respectively; *n* = 6 animals for each group. **(B)** RT-PCR to quantitate the expression of *α-SMA*, *fibronectin*, and *Col1a1* in kidney tissues; *n* = 6 animals for each group. **(C)** Western blot to detect the protein expression of α-SMA, fibronectin, and Col1a1. The right graph is the quantitation; *n* = 3 animals for each group. **(D)** Representative images of IHC show the tissue expression of α-SMA and Col1a1, respectively. The right graphs are quantitation of the percentage of α-SMA–positive and Col1a1-positive areas, respectively; *n* = 6 animals for each group. **(E)** RT-PCR to quantitate the expression of let-7i-5p in kidney tissues; *n* = 4 animals in each group. ∗∗*p* < 0.01 and ∗∗∗*p* < 0.001 *versus* sham; *n* = 5 animals for each group.

As shown in Figure 1, mice with UUO operation presented dilated tubular lumen, expanded interstitial area, and fibrosis as revealed by HE and Masson staining (Figure 1A). Accordingly, the expression of fibrogenic genes α-SMA, fibronectin, and Col1a1 was significantly increased in the kidneys after UUO as detected by RT-PCR, Western blot, and immunohistochemistry (Figures 1B–D). Notably, accompanying the tubular injury and interstitial fibrosis, we also detected significantly elevated expression of let-7i-5p in the kidneys after UUO (Figure 1E).

### Knockdown of let-7i-5p ameliorated UUO-induced renal fibrosis

**FIGURE 2:**
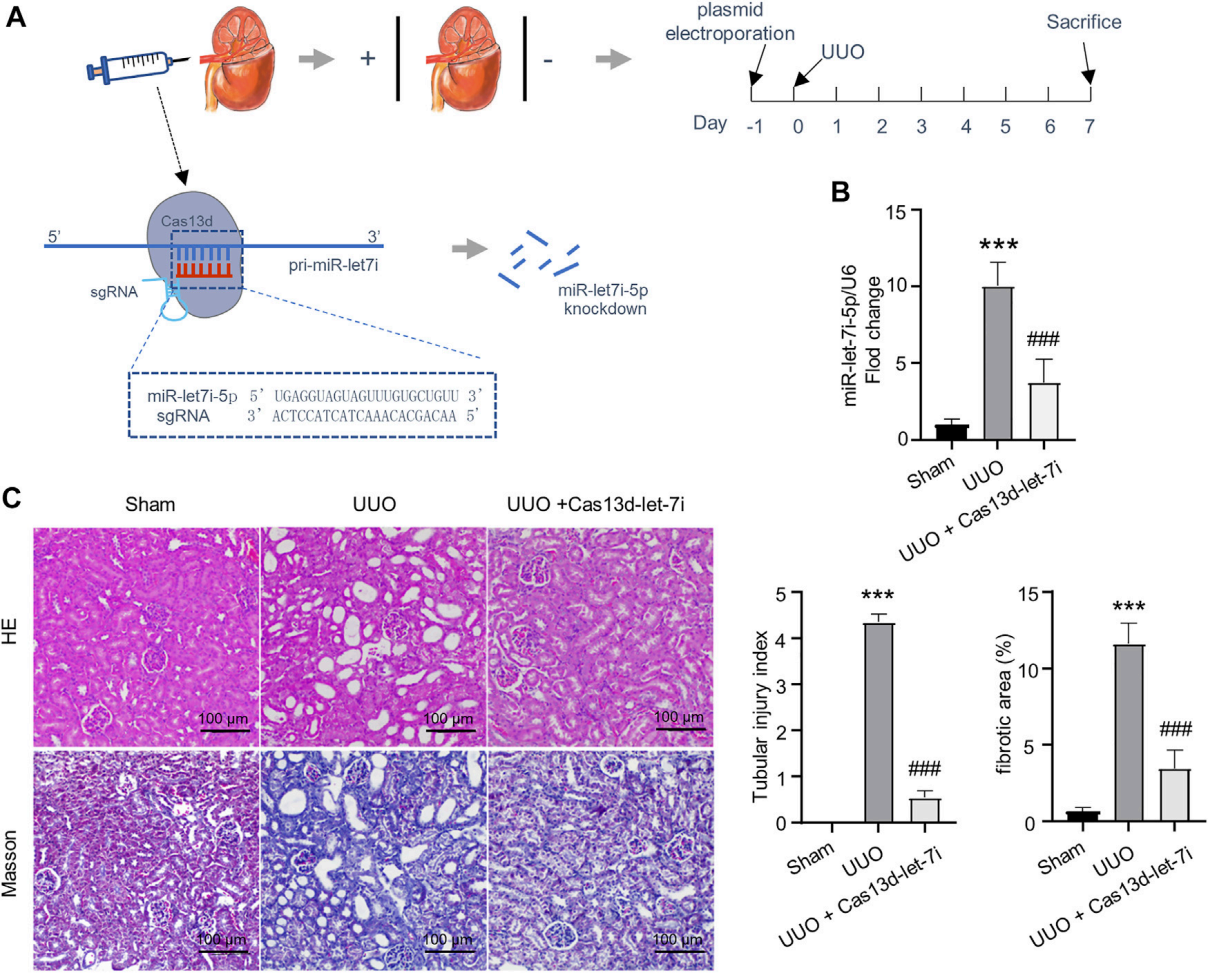
Knockdown of let-7i-5p reduces renal fibrosis in the kidney after UUO. **(A)** Schematic graph illustrates the experimental design of electroporation of miR-let-7i–targeting pCas13d-let-7i plasmid in the kidney after UUO. 100 μg of pCas13d-let-7i plasmid was electroporated into the kidney one day before UUO operation. 7 days post–UUO operation, the kidney tissue was harvested for analysis. **(B)** RT-PCR to validate the efficiency of let-7i-5p knockdown by pCas13d-let-7i electroporation. *n* = 4 animals for sham and UUO groups. *n* = 6 animals for the group of UUO + Cas13d-let-7i. **(C)** Histopathological analysis of kidney tissue by HE and Masson staining, and the corresponding quantitation of the tubular injury index and fibrotic areas. *n* = 4 animals for each group. ∗∗∗*p* < 0.001 *versus* sham and ^###^ *p* < 0.001 *versus* UUO.

**FIGURE 3:**
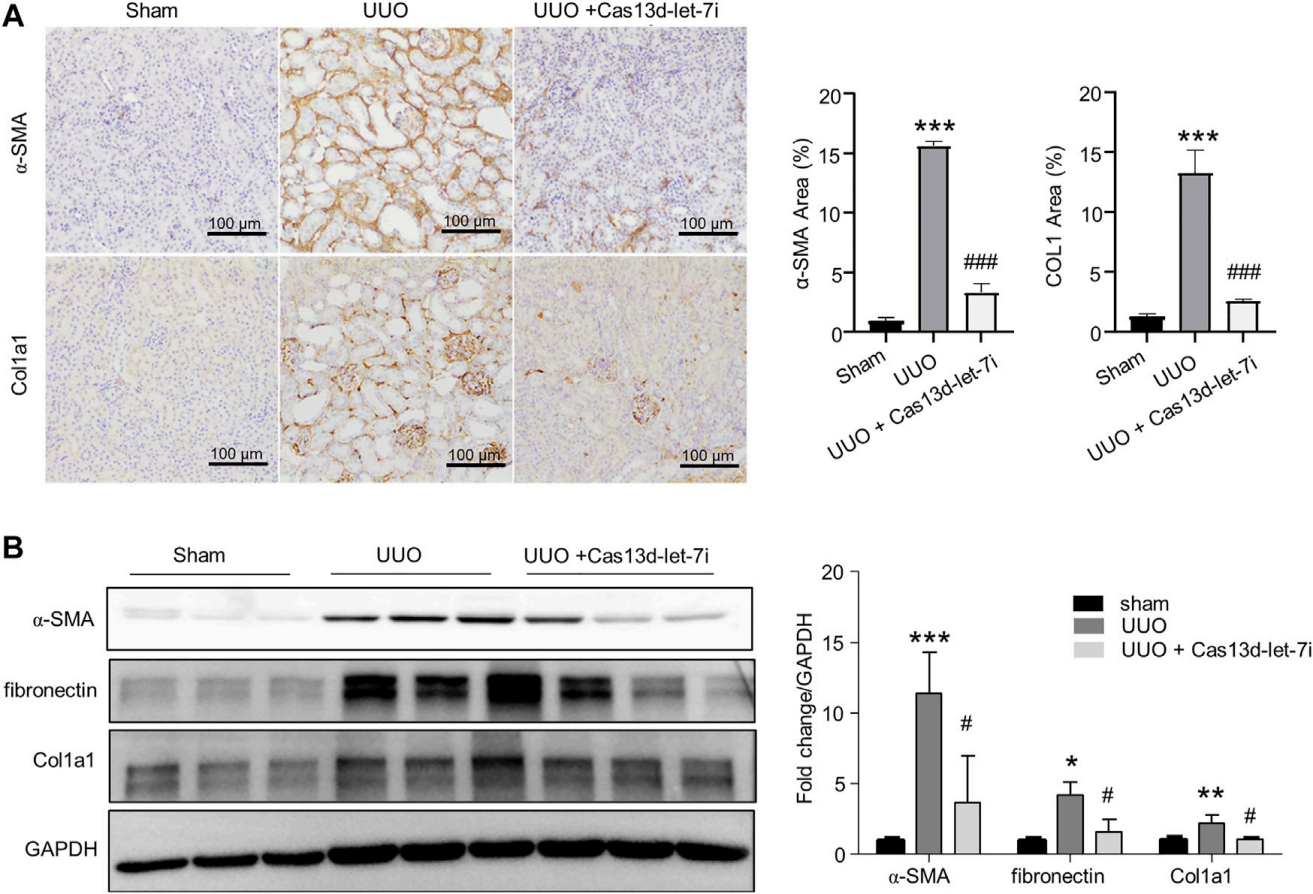
Knockdown of let-7i-5p suppresses the expression of fibrotic genes in the kidneys after UUO. **(A)** Representative IHC images of α-SMA and Col1a1 along with their quantitative analysis. *n* = 4 animals for sham and UUO groups. *n* = 6 animals for the group of UUO + Cas13d-let-7i. **(B)** Western blot to show the expression of α-SMA, fibronectin, and Col1a1. *n* = 3 animals for each group. ∗*p* < 0.05, ∗∗*p* < 0.01, and ∗∗∗*p* < 0.001 *versus* sham. ^#^ *p* < 0.05 and ^###^ *p* < 0.001 *versus* UUO.

The CRISPR/Cas13d system is a state-of-the-art RNA editing technology that can realize efficient RNA knockdown with high specificity both *in vitro* and *in vivo* (Konermann et al., 2018). On the other hand, it has been reported that local gene delivery into the kidneys can be achieved by electroporation (Isaka, 2006). To analyze the function of let-7i-5p in UUO-induced renal fibrosis, we constructed a vector-expressing CRISPR/Cas13d and gRNA targeting pri-miR-let-7i (pCas13d-let-7i). The vector was retrogradely injected into the kidneys *via* the renal vein followed by electroporation, as described in the Materials and Methods section, 1 day before the UUO operation (Figure 2A). RT-PCR showed that electroporation of the pCas13d-let-7i plasmid can significantly knock down let-7i-5p expression in the kidneys after UUO (Figure 2B). Importantly, knockdown of let-7i-5p also notably alleviated tubular dilation and interstitial area expansion with decreased tubular injury index as revealed by HE staining (Figure 2C). Masson staining revealed obviously attenuated interstitial fibrosis in the kidneys after UUO with let-7i-5p knockdown (Figure 2C). In accordance, knockdown of let-7i-5p also reduced the immunostaining of α-SMA and Col1a1 in the kidneys after UUO (Figure 3A). Western blot of the kidney lysate showed significantly decreased expression levels of α-SMA, fibronectin, and Col1a1 in the kidneys after UUO with let-7i-5p knockdown (Figure 3B). These data suggest that let-7i-5p promotes renal fibrosis in the kidneys after UUO.

### Knockdown of let-7i-5p blunted TGF-β1–induced fibrosis in renal tubular cells

**FIGURE 4:**
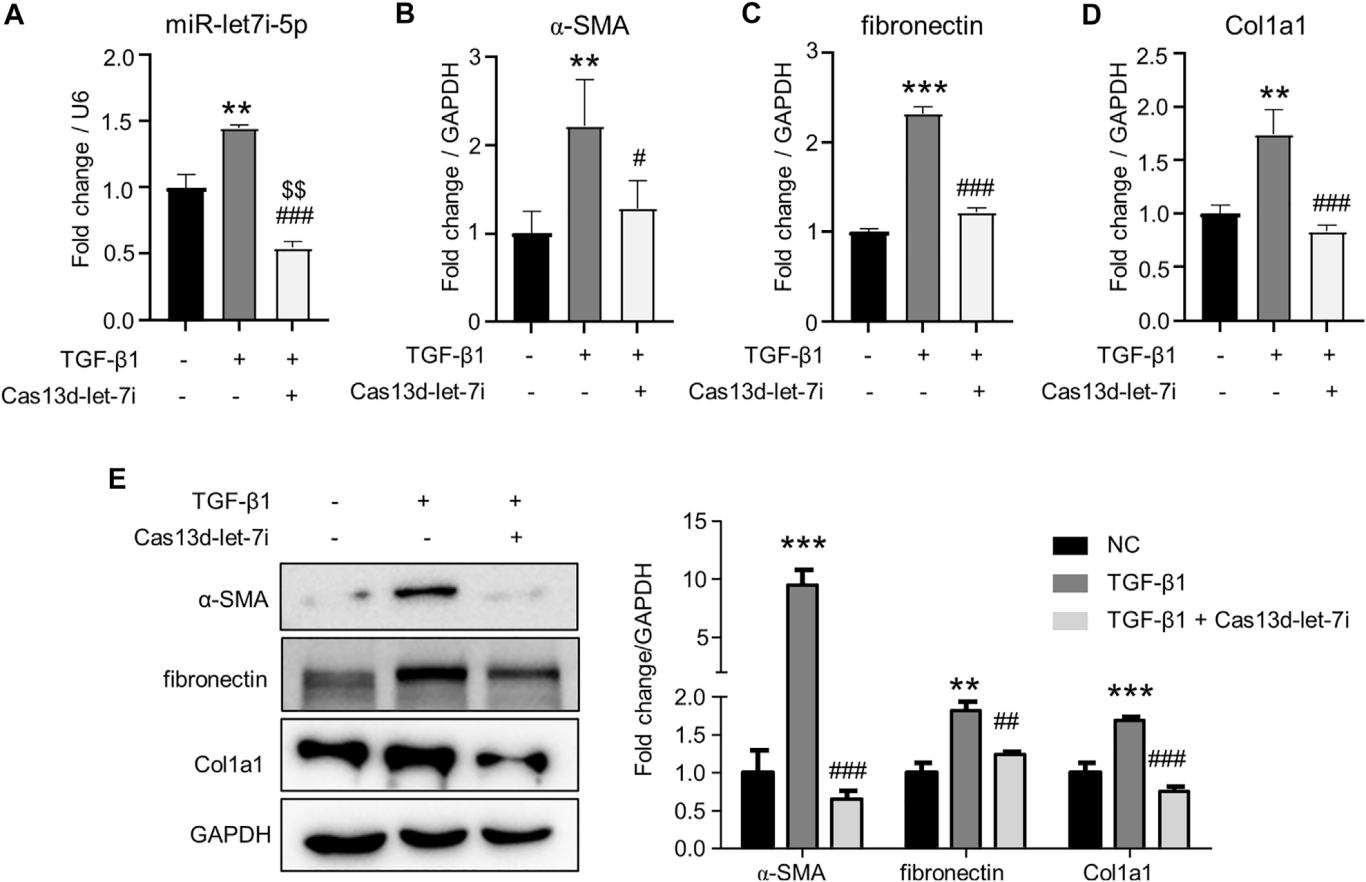
Knockdown of let-7i-5p attenuates fibrosis in TGF-β1–treated TCMK1 cells. The TCMK1 cells were transfected with pCas13d-let-7i plasmid (Cas13d-let-7i) for 12 h. Then, the cells were starved in low-serum (0.5%) medium for 36 h. In the last 24 h, 5 ng/ml TGF-β1 was supplemented. RNA was isolated for the detection of let-7i-5p **(A)**, *α-SMA* **(B)**, *fibronectin* **(C)**, and *Col1a1* **(D)** by RT-PCR. Western blot was also used to detect the protein levels of α-SMA, fibronectin, and Col1a1 **(E)**. Each treatment was performed in triplicates. ∗∗*p* < 0.01 and ∗∗∗*p* < 0.001 *versus* NC. ^#^ *p* < 0.05, ^##^ *p* < 0.01, and ^###^ *p* < 0.001 *versus* TGF-β1. ^$$^ *p* < 0.01 versus NC.

We further analyzed the role of let-7i-5p in renal fibrosis in TGF-β1-treated renal tubular cell TCMK1. Cas13d was again used to knock down let-7i-5p expression by the transfection of pCas13d-let-7i. As shown in Figure 4A, TGF-β1 treatment upregulated let-7i-5p expression. Notably, transfection of the pCas13d-let-7i vector significantly reduced let-7i-5p expression when compared with TGF-β1–treated or non-treated TCMK1 cells. Importantly, TGF-β1 induced the transcription of *α-SMA*, *fibronectin*, and *Col1a1* which were suppressed by let-7i-5p knockdown (Figures 4B–D). Furthermore, Western blot showed that let-7i-5p knockdown reduced the protein levels of α-SMA, fibronectin, and Col1a1 in TGF-β1–treated TCMK1 cells (Figure 4E). Collectively, these *in vivo* and *in vitro* findings definitely prove that let-7i-5p is a profibrotic miRNA in renal fibrosis.

### Let-7i-5p is a target gene of TGF-β/Smad3 pathway

**FIGURE 5:**
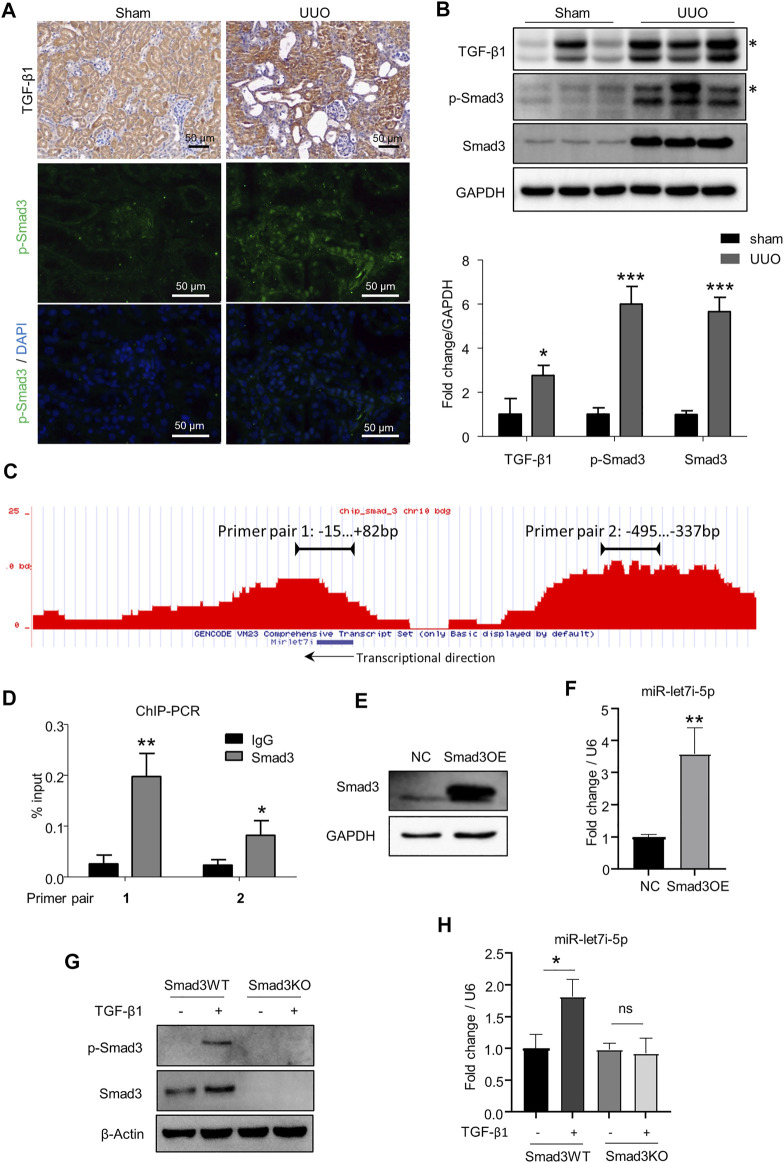
Let-7i-5p functions as a target gene of TGF-β/Smad3 signaling. **(A,B)** Expressions of TGF-β1 and p-Smad3 were detected by IHC, fluorescent immunostaining, and Western blot in kidney tissue. The asterisk symbols in B indicate an unspecific band. **(C)** ChIP-seq to reveal the binding enrichment by Smad3 in *miR-let-7i* locus in TCMK1 cells. The arrow indicates the transcriptional direction. **(D)** ChIP-PCR was performed to validate the binding of Smad3 to the *miR-let-7i* locus. The amplification regions of the two primer pairs were labeled in **(B)**. ∗*p* < 0.05 and ∗∗*p* < 0.01 *versus* IgG. **(E)** TCMK1 cells were transfected with Smad3-expressing plasmid (Smad3OE). 48 h later, Western blot was used to detect Smad3 expression. **(F)** Expression of let-7i-5p analyzed by RT-PCR in TCMK1 cells with Smad3 overexpression. ∗∗*p* < 0.01 *versus* NC. **(G)** Smad3 knockout TCMK1 cell clone (Smad3KO) and its wild-type counterpart (Smad3WT) were starved for 12 h in the low-serum medium followed by 5 ng/ml TGF-β1 stimulation for another 12 h. The expression levels of Smad3 and p-Smad3 were checked by Western blot. **(H)** Expression of let-7i-5p was checked in Smad3WT and Smad3KO TCMK1 cells treated with TGF-β1, the same as in **(F)**. ∗*p* < 0.05. ns, no significance. In **(D, F, H)**, assays were performed in triplicates.

TGF-β signaling plays a pivotal role in renal fibrosis, and Smad3 is the dominant downstream transcriptional factor controlling the expression of various pro-fibrogenic genes (Wang et al., 2021). Consistently, TGF-β/Smad3 signaling was highly activated in the kidneys after UUO as revealed by the increased expression of TGF-β1 and activation of Smad3 (p-Smad3) (Figures 5A,B). By ChIP-seq analysis in TCMK1 cells, we were surprised to find two binding peaks by Smad3 in the chromatin regions surrounding the let-7i locus including the possible 5′ cis-regulatory element, coding region, and 3′ flanking region (Figure 5C). The binding of Smad3 to the above two sites on the let-7i genomic locus was further confirmed by ChIP RT-PCR with corresponding primers (primer pairs 1 and 2 in Figure 5D). This suggests that the expression of let-7i may be directly regulated by Smad3. To explore this possibility, we overexpressed Smad3 in TCMK1 cells by transfecting a Smad3-expressing plasmid (Figure 5E). As shown in Figure 5F, overexpression of Smad3 directly promoted let-7i-5p transcription. We further established a Smad3 knockout (Smad3KO) TCMK1 cell clone by the CRISPR/Cas9-mediated genomic edition. The Smad3KO TCMK1 cells showed complete deletion of Smad3 expression and TGF-β1–stimulated Smad3 phosphorylation (Figure 5G). Importantly, the deletion of Smad3 abolished TGF-β1–stimulated upregulation of let-7i-5p (Figure 5H). These data have confirmed that let-7i-5p is a target gene downstream of the TGF-β/Smad3 pathway.

## Discussion

Chronic fibrosis is an important feature of all types of CKD. MicroRNAs play pivotal roles in renal fibrosis (Wang et al., 2021). In this study, we showed that microRNA let-7i-5p was upregulated in the kidneys after UUO and TGF-β1–treated renal tubular cell line TCMK1. Knockdown of let-7i-5p attenuated the fibrotic phenotype with decreased expression of α-SMA, fibronectin, and Col1a1 and improved kidney histology in the kidneys after UUO. Furthermore, knockdown of let-7i-5p also decreased TGF-β1–induced fibrosis in TCMK1 cells. These functional findings suggest that let-7i-5p is a pathogenic microRNA to promote renal fibrosis. Therefore, our results coincide with those of Jin et al. (2021) and Sun et al. (2021) who also reported a pro-fibrotic role of let-7i-5p in CKD.

One important novelty of this study is the finding that let-7i-5p is a target miRNA directly regulated by the well-established pro-fibrotic TGF-β/Smad3 pathway. Considerable studies have reported the target genes regulated by let-7i-5p in several fibrotic diseases including renal fibrosis (as discussed below). Nevertheless, little is known about the mechanism regulating let-7i-5p expression. TGF-β signaling promotes renal fibrosis in CKD primarily through the downstream transcriptional factor Smad3 (Wang et al., 2021). It has been reported that many microRNAs take part in renal fibrosis and are regulated in a Smad3-dependent manner (Wang et al., 2021). By advantage of ChIP-seq in renal tubular cells, we found that Smad3 can bind to the genomic locus of let-7i. Overexpression of Smad3 can directly promote the transcription of let-7i-5p. Furthermore, TGF-β1 stimulates let-7i-5p expression in a Smad3-dependent way as deletion of Smad3 in TCMK1 cells abolished TGF-β1–induced let-7i-5p expression. Therefore, let-7i-5p is a target gene of the TGF-β/Smad3 pathway. Thus, our findings for the first time reveal a novel mechanism underlying the regulation of let-7i-5p in renal fibrosis.

It is reported that let-7i-5p promotes renal fibrosis by targeting tuberous sclerosis complex subunit 1 (*Tsc1*) to activate the mammalian target of rapamycin (mTOR) signaling in UUO mice (Jin et al., 2021). Furthermore, another study by Sun et al. (2021) showed that let-7i-5p targets polypeptide N-acetylgalactosaminyltransferase 1 (*Galnt1*) to promote renal fibrosis in UUO mice and folic acid–induced CKD mice. In contrast to the pro-fibrotic effect on renal disease, let-7i-5p seems to play an anti-fibrotic role in the fibrotic diseases of the heart, vasculature, and lungs (Wang et al., 2015; Chen et al., 2020; Xu et al., 2022). In hypertensive cardiac fibrosis, let-7i-5p suppresses fibrosis by targeting *Col1a2*, *Col3a1*, *Col4a1*, and *Col5a2* (Wang et al., 2015). In vascular and pulmonary fibrosis, let-7i-5p inhibits endothelial to mesenchymal transition (EndoMT) or fibroblast activation by targeting *Tgfbr1* (Chen et al., 2020; Xu et al., 2022). These interorgan discrepancies imply that let-7i-5p shows disease-dependent effects on fibrosis. A similar paradoxical role of let-7i-5p on cell proliferation was also reported in that let-7i-5p suppresses cardiomyocyte proliferation but promotes renal tumor cell proliferation (Hu et al., 2019; Liu et al., 2021). Therefore, it would be interesting to clarify the mechanism underlying these contrary and disease-dependent functions of let-7i-5p in future work.

CRISPR/Cas13d is thought to be a powerful tool to knock down RNA (Konermann et al., 2018). As the target recognition of Cas13d does not require the flanking protospacer sequence, sgRNA can be designed to target any specific region across the entire RNA sequence. On the other hand, it is reported that the selected RfxCas13d can knock down target RNA more efficiently and specifically than the conventional RNA interference technologies (Konermann et al., 2018). In this study, we combined the CRISPR/Cas13d system with the established *in situ* organ electroporation technology to realize the efficient knockdown of endogenous microRNA *in vivo*. Together with the knockdown study in TCMK1 cells, our results suggest that the CRISPR/Cas13d is a promising technology for the rapid functional study of microRNA *in vitro* and *in vivo*.

Conclusively, findings in this study demonstrate that let-7i-5p is a pro-fibrotic microRNA and is regulated by the TGF-β/Smad3 pathway in renal fibrosis.

## Data Availability

The raw data for ChIP-seq has been deposited in the Gene Expression Omnibus database (https://www.ncbi.nlm.nih.gov/geo/, Accession Number GSE203063).
