# Supplementary material for: The Smad3-dependent microRNA let-7i-5p promoted renal fibrosis in mice with unilateral ureteral obstruction
Source: Front Physiol. 2022 Aug 25;13:937878. doi: 10.3389/fphys.2022.937878 (PMC9452756; doi:10.3389/fphys.2022.937878)
Supplement: Supplementary file 1 [file DataSheet1.docx]

Supplementary Material

# Supplementary Data

The raw ChIP-seq data can be downloaded from the Gene Expression Omnibus database with the accession number of GSE203063.

# Supplementary Figures and Tables

## Supplementary Figures


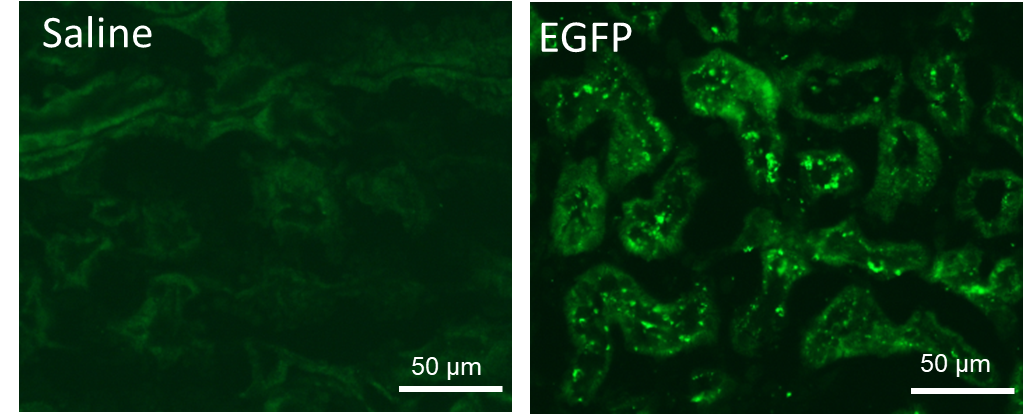


**Supplementary Figure 1.** Electroporation of an EGFP-coding plasmid to monitor the gene delivery efficiency in kidney. 100 μg of pEGFP-C1 plasmid or equal volume of saline was retrogradely injected into kidney followed by electroporation. 3 days post-electroporation, cryosection of the kidney was checked for EGFP expression under a fluorescent microscope.

## Supplementary Figures

**Supplementary Table 1. Primers used in this study.**

| **Primer name** | **Forward / Reverse (5’ to 3’)** | **Application** |
| --- | --- | --- |
| a-SMA | CCCAGACATCAGGGAGTAATGG | RT-PCR |
|  | TCTATCGGATACTTCAGCGTCA |  |
| fibronectin | ATGTGGACCCCTCCTGATAGT | RT-PCR |
|  | GCCCAGTGATTTCAGCAAAGG |  |
| Col1a1 | ATCCAACGAGATCGAGCTCA | RT-PCR |
|  | AAGGGAGCCACATCGATGAT |  |
| GAPDH | GGTGAAGGTCGGTGTGAACG | RT-PCR |
|  | CTCGCTCCTGGAAGATGGTG |  |
| miR-let7i-5p | CGCGCGTGAGGTAGTAGTTTGT | RT-PCR |
|  | AGTGCAGGGTCCGAGGTATT |  |
| U6 | GCTCGCTTCGGCAGCACATATAC | RT-PCR |
|  | CGAATTTGCGTGTCATCCTTGCG |  |
| Chip.let7i.primer pair 1 | GGCTGAGGTAGTAGTTTGTG | ChIP-PCR |
|  | TGAGCATCACCAGCACTAGCA |  |
| Chip.let7i.primer pair 2 | GTCAGAACAGTCAACGCTG | ChIP-PCR |
|  | GTCAACCCTGTTTAAGGAG |  |

**Supplementary Table 2. List of antibodies used in this study.**

| **Antibody** | **Company** | **Catalog No.** | **Application** |
| --- | --- | --- | --- |
| Primary antibody | | | |
| Rabbit anti-Phospho-Smad3 (Ser 423/425) | Cell Signaling, USA | 9520 | IF |
| Rabbit anti-Smad3 | Cell Signaling, USA | 9523 | WB, ChIP |
| Rabbit anti-TGF-β1 | Boster, China | BA0290 | IHC |
| Rabbit anti-α-SMA | Proteintech, USA | 14395-1 | F-IHC, WB |
| Rabbit anti-fibronectin | Abmart, China | T59537 | WB |
| Rabbit anti-Col1a1 | Abmart, China | TA7001 | F-IHC, WB |
| Rabbit anti-GAPDH | Abways, China | AB0037 | WB |
| Secondary antibody | | | |
| Donkey anti-Rabbit IgG, Alexa Fluor 488 | Thermo Scientific, USA | A21206 | F-IHC |
| HRP-conjugated goat anti-rabbit IgG | Sangon, China | D110058 | WB |

Note: ChIP, chromatin immunoprecipitation. WB, Western blot. IHC, immunohistochemistry. F-IHC, fluorescent immunohistochemistry.
